# Using wet-bulb globe temperature meters to examine the effect of heat on various tennis court surfaces

**DOI:** 10.1038/s41598-024-66518-8

**Published:** 2024-07-05

**Authors:** Hiroki Yamaguchi, Takaaki Mori, Hiromi Hanano, Kan Oishi, Kentaro Ikeue, Yuiko Yamamoto, Kojiro Ishii

**Affiliations:** 1 Graduate School of Health and Sports Science, Doshisha University, Kyotanabe, Kyoto Japan; 2 Japan Society for the Promotion of Sciences, Chiyoda-ku, Tokyo Japan; 3 Department of Physical Activity Research, National Institutes of Biomedical Innovation, Health and Nutrition, Ibaraki, Osaka Japan; 4 Department of Endocrinology, Clinical Research Institute, Metabolism, and Hypertension Research, National Hospital Organization Kyoto Medical Center, Kyoto, Kyoto Japan; 5 Faculty of Health and Sports Science, Doshisha University, Kyotanabe, Kyoto Japan

**Keywords:** Wet-bulb globe temperature, Tennis court surface, Thermal environment, Heat-related illness, Climate sciences, Environmental sciences, Health care, Disease prevention

## Abstract

In this study, we evaluated the thermal environments of different tennis courts using wet-bulb globe temperature (WBGT) meters. WBGT meters were installed in an outdoor hard court, sand-filled artificial grass court, and clay court (a softball field), and measurements were taken hourly from 9:00 to 17:00 on weekdays from June 1 to September 21, 2022. The results were compared with data from different courts and the nearest Japan Meteorological Agency station (JMA WBGT) based on the Japan Sports Association’s guidelines for exercise to prevent heat stroke. The median WBGT on each court was significantly higher for hard courts at the “Warning” (25 ≤ JMA WBGT < 28) level or above, sand-filled artificial grass courts at the “Severe Warning” (28 ≤ JMA WBGT < 31) level or above, and clay courts at the “Danger” (31 ≤ JMA WBGT) level than the JMA WBGT. Compared with the JMA WBGT, hard and sand-filled artificial grass courts are played on under particularly hot conditions. The results of this study could indicate to tournament organizers and coaches the importance of measuring the WBGT on each court surface from an early stage to prevent heat-related incidents.

## Introduction

In tennis competitions, multiple matches are played on a single day, lasting as long as five hours^1^. Moreover, among the four major tournaments designated by the International Tennis Federation (ITF), the temperatures at the Australian Open and US Open can exceed 35 °C. Furthermore, the Tokyo 2020 Olympic Games (Tokyo Olympics) in 2021 faced extremely hot conditions, with many players calling for adequate countermeasures^2–4^. Consequently, the ITF formulated and issued the “Extreme Weather Policy” at the Tokyo Olympics to provide guidelines for match management based on the temperature^5^. However, heat-related illnesses, such as heatstroke, are still considered a major problem in tennis.

Tennis matches and practices are played on various court surfaces. In the 2022 junior division of international tournaments organized by the ITF, 437 tournaments (55.7%) were played on hard courts, 323 (41.2%) on clay courts, 20 (2.6%) on carpeted courts (including sand-filled artificial grass courts), and four (0.5%) on natural grass courts. Hard and clay courts account for most of those used in international tournaments^6^. However, among the junior international, national, and major qualifying tournaments in Japan, 74 tournaments (71.2%) were played on sand-filled artificial grass courts (including three tournaments on artificial grass courts), 23 (22.1%) on hard courts, and seven (6.7%) on clay courts in 2022, with sand-filled artificial grass courts accounting for approximately 70%^7^. Sand-filled artificial grass courts are characterized by low stress on the legs and hips, good drainage, and ease of maintenance, even in light rain. Three types of surfaces are used in Japan: hard, sand-filled artificial grass and clay courts.

During matches and practices in hot environments, players are subjected to thermal loads from solar radiation, reflected solar radiation, and ground surface radiation^8^. These effects can differ depending on the surface material being used. Previous studies have reported that different playing surfaces and surrounding environments constitute diverse microclimatic environments^9,10^ that may differ from established weather stations^11^. The wet-bulb globe temperature (WBGT) is an index used to measure the thermal environment calculated from wet-bulb, black-bulb, and dry-bulb temperatures and was proposed by Yaglow and Minard in 1957^12^. The WBGT has been reported to be highly correlated with heat stroke^13^ and can be used to prevent it. The WBGT was ISO-approved in 1989^14^, and the Japan Sports Association published the Heat Stroke Prevention Exercise Guidelines in 1994 in Japan^15^, presenting measures to prevent heat stroke in accordance with the WBGT. The WBGT is currently used in the thermal environment assessment of international sporting events such as the Olympic Games^16^, and an accurate understanding of the thermal environment using WBGT measurements at each sports field has become a fundamental element of heat countermeasures. In tennis, heat safety guidelines—such as the Extreme Weather Policy^5^ and the Japan Tennis Association (JTA)’s heat rules^17^—are operated based on the WBGT.

Moreover, several studies have been conducted on the differences in the WBGT on various athletic surfaces. Pryor et al.^11^ evaluated the WBGT for various stadiums using portable WBGT meters and reported that no differences in the WBGT were evident for each surface but that there was considerable variation in the WBGT between surfaces and that using the WBGT published by the National Weather Service misclassified heat safety categories for all surfaces. Grundstein et al.^10^ also compared the WBGT measured on grass, artificial turf, and tennis hard courts and reported no differences in the WBGT between surfaces, although microclimate differences in dry-bulb and dew-point temperatures were evident. However, in the study by Pryor et al.^11^, the time of measurement was limited to the afternoon (between 13:00 and 16:30), and it was not clear from which guideline level the WBGT error at the station was created at each surface. The study by Grundstein et al.^10^ also has several limitations. They did not make comparisons for each exercise guideline category, nor did they perform measurements on clay courts, nor did they compare the WBGT values to those at the nearest station. Consequently, based on the above problems, the investigation of the heat conditions at various tennis courts during the summer season could provide data to use as a reference for exercise guidelines and playing time on each surface. Moreover, long-term measurements for the duration of summer competitions could contribute to effective heat management for athletes, coaches, referees, and spectators. Therefore, this study aims to evaluate the heat environments of different tennis court surfaces using WBGT meters.

## Methods

### Measurement environment and the WBGT device

Natural wet-bulb and dry-bulb, and 15-mm-diameter black-bulb (AGTS-2505, Ando Keiki, Tokyo, Japan; CK-SETII-NM, Ando Keiki) WBGT meters (natural thermometers) were installed approximately 1.5 m above the surface of a blue-painted hard court (DecoTurf, Sports Surface, Tokyo, Japan), sand-filled artificial grass court (sandgrass, Sekisui Jushi Corporation, Tokyo), and clay court (decomposed granite soil) (softball field) at Doshisha University in Kyotanabe, Kyoto, Japan. Measurements were taken every hour from 9:00 to 17:00 on weekdays between June 1, 2022, and September 21, 2022. The dry-bulb temperature was measured in the shade, with a temperature-sensitive area covered with aluminum foil to remove radiant heat. Ethical approval was not required for this study.

### Collection of WBGT data

The dry-bulb, wet-bulb, and black-bulb temperatures were recorded, and the WBGT was calculated using the outdoor WBGT calculation formula (0.7 × wet-bulb temperature + 0.2 × black-bulb temperature + 0.1 × dry-bulb temperature) (hard WBGT, sand-filled artificial grass WBGT, clay WBGT). The values published by public organizations are based on the Electronic Information Provision Service for Heat Index (WBGT) predictions^18^ (provisioning service) of the Ministry of the Environment according to the observed values of the Japan Meteorological Agency (JMA). In its Application of Heat Rules and Medical Rules in Tennis Competitions in Heat publication of 2019, the JTA requires that the WBGT at the nearest point to the tournament venue be checked every hour using a provisioning service whenever the WBGT on-site measurements are not available^17^. Consequently, this study recorded the estimated WBGT (JMA WBGT) at the station nearest the measurement location (Kyotanabe). The JMA WBGT was calculated based on the work of Ono and Tonouchi using the following equation^19^:

$$\begin{aligned} JMA WBGT = & 0.735 \times Ta + 0.0374 \times RH + 0.00292 \times Ta \times RH \\ & + 7.619 \times SR - 4.557 \times SR2 - 0.0572 \times WS - 4.064 \\ \end{aligned}$$

where *Ta* denotes the temperature (°C), *RH* denotes the relative humidity (%), *SR* denotes the total solar radiation (kW/m^2^), and *WS* denotes the average wind speed (m/s).

In the study^19^, the black-bulb temperature (measured using a 6-inch black-bulb thermometer), dry-bulb temperature, and wet-bulb temperature (calculated by Iribarne and Godson, 1981^20^ using the dry-bulb temperature, dew point temperature, and ground pressure) were used to calculate the WBGT reference values. The WBGT estimation equation was calculated by multiple regression using the normally observed meteorological elements—that is, the dry-bulb temperature, relative humidity, dry-bulb temperature × relative humidity, total solar radiation, and wind speed—as explanatory variables. It should be noted that the infrared radiation is not measured in the JMA’s normally observed meteorological elements, and the total solar radiation (which is the sum of direct and scattered solar radiation) is observed.

The correlation between the estimated and observed values was extremely high (r^2^ = 0.998), and 97.6% of the estimation error was within ± 1 °C. The quadratic terms of the total solar radiation (which showed estimation errors between the estimated and observed values) were then added as explanatory variables. The results showed that 99.8% of the estimation errors were within ± 1 °C, 92.7% were within ± 0.5 °C, and 33.0% were within ± 0.1 °C. The three-year data range for the six weather stations used to calculate the equation ranged from − 3.9 °C to 38.2 °C for the temperature, 44% to 92% for temperatures below 0 °C and 28% to 52% for temperatures above 35 °C for the relative humidity.

Consequently, this formula can be used to estimate the WBGT for six cities over three years with a confidence level of 98.3–99.8% and a bias within 1.0 °C and is currently used by the JMA as the WBGT estimation formula in Japan. Global solar radiation and humidity were calculated based on values from neighboring regional meteorological stations, as 687 stations nationwide (approximately 82% of all stations in Japan), including the Kyotanabe station, do not have actual measured values. It should be noted that the use of the WBGT at the nearest station, published by a public agency for comparison with the measured WBGT has been used in a previous study^11^ that investigated the thermal environment at various stadiums. All the measurement points on the three court surfaces were within approximately 100–200 m of each other. The distance between the tennis courts and Kyotanabe Station was approximately 3.8 km.

### Statistical analysis

The hard court WBGT, sand-filled artificial grass court WBGT, clay court WBGT, and JMA WBGT were compared based on the guideline levels for heat stroke prevention (21 ≤ JMA WBGT < 25: Caution, 25 ≤ JMA WBGT < 28: Warning, 28 ≤ JMA WBGT < 31: Severe Warning, 31 ≤ WBGT: Danger) published by the JSA^15^. The limit values for each guideline level were determined based on an analysis of the distribution of ambient temperatures (WBGT) during the occurrence of heat stroke during exercise in Japan. The Steel–Dwass test was used to compare the WBGT and JMA WBGT for each surface. A Bland–Altman analysis was conducted for the WBGT of each surface and the JMA WBGT to confirm systematic errors^21^. Fixed errors were considered to exist in the difference between the WBGT of each surface and the JMA WBGT if the range of ± 1.96 standard deviation from the mean (which was the limit of agreement) did not reach zero. Proportional errors were determined by regression analysis on the Bland–Altman plots, and proportional errors were considered to exist when the regression was significant. Hourly comparisons were made between the WBGT of each surface and the JMA WBGT using the Steel–Dwass test. R software ver. 4.2.2 and SPSS software ver. 28.0 (IBM Japan, Tokyo, Japan) were used for the statistical analyses. Statistical significance was set at *P* < 0.05.

### Human and animal rights

Ethical approval and informed consent were not required for this study as it did not involve human subjects.

## Results

We collected data for 417 sets of data, excluding 322 sets of data when the WBGT meter failed, when it rained, or when the court could not use or record (WBGT meter failure: 33 sets of data, rain: 135 sets of data, the court could not use or record: 154 sets of data).

### Comparison of the WBGT of each surface and the JMA WGBT

**Figure 1:**
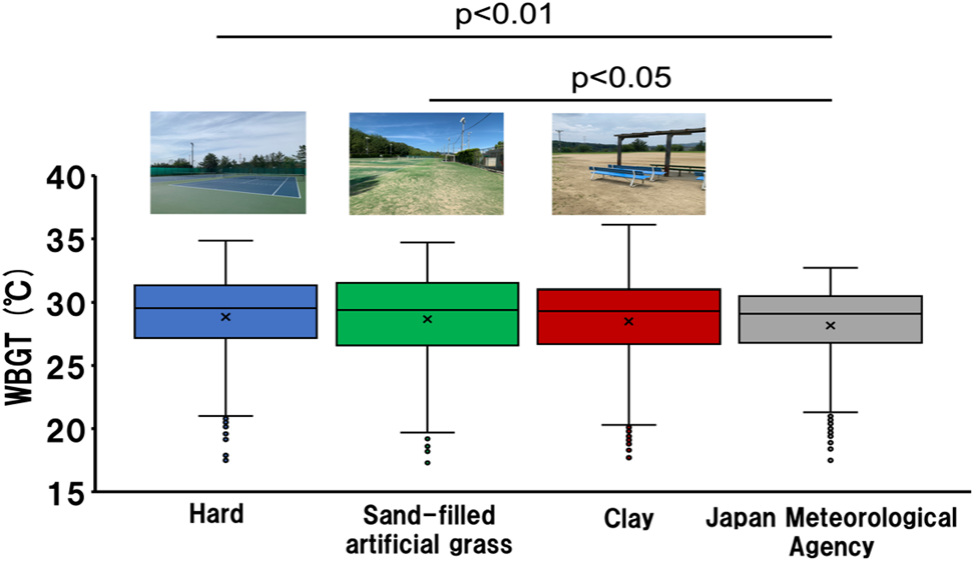
Comparative WBGT among different court surfaces (n = 417).

Figure 1 shows a comparison of the WBGT for different court surfaces. The hard court WBGT and sand-filled artificial grass court WBGT are significantly higher than the JMA WBGT (hard WBGT, median: 28.9 °C [first quartile: 27.2 °C; third quartile: 31.3 °C]; sand-filled artificial grass WBGT, median: 28.7 °C [first quartile: 26.6 °C; third quartile: 31.5 °C]; and JMA WBGT, median: 28.2 °C [first quartile: 26.8 °C; third quartile: 30.5 °C]; hard WBGT, *p* < 0.01; sand-filled artificial grass WBGT, *p* < 0.05) when all recorded data are compared.

**Figure 2:**
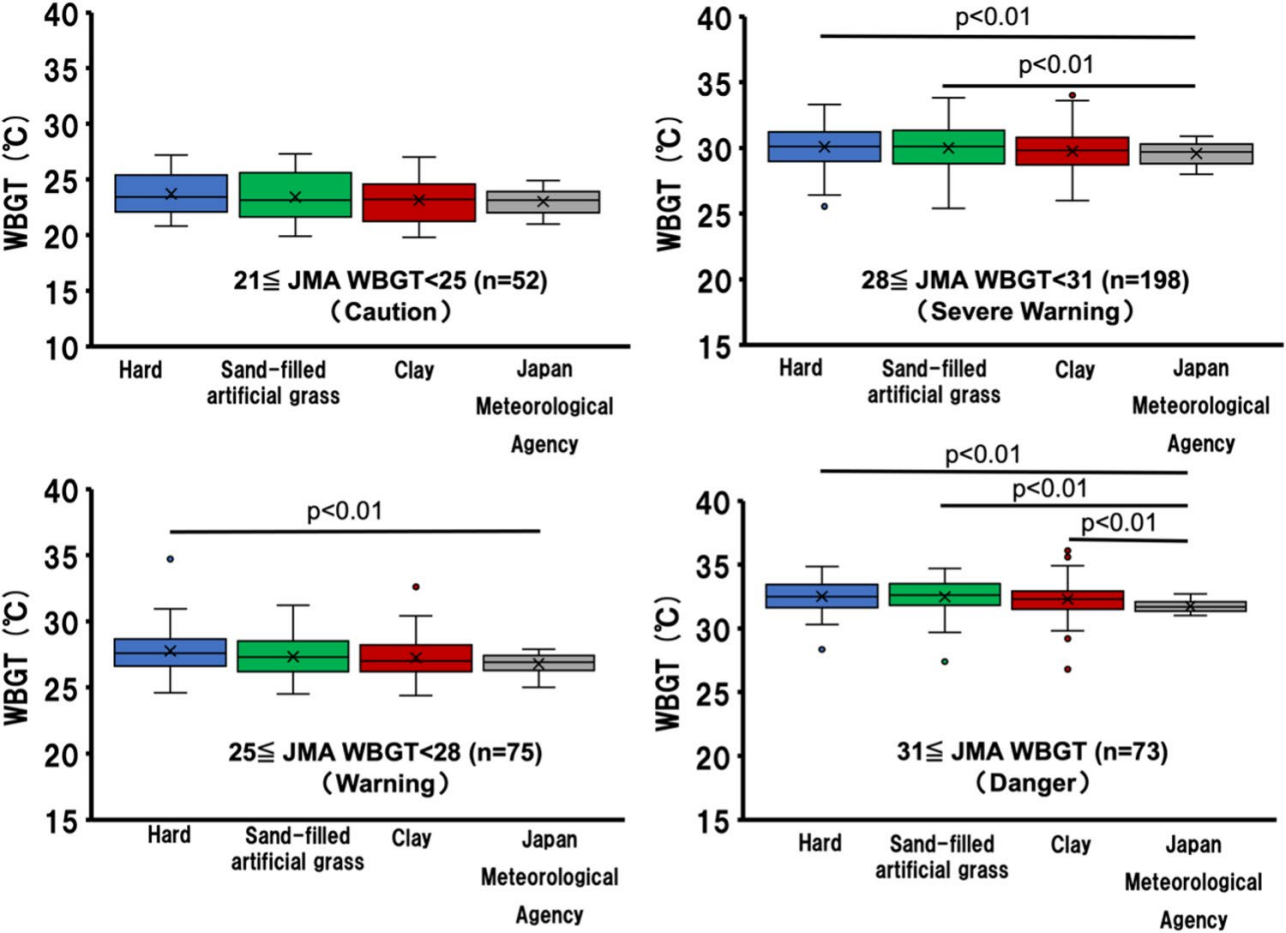
Comparative WBGT among different court surfaces based on exercise guideline levels.

Figure 2 shows the comparative WBGT among different court surfaces based on JSA exercise guideline levels (guideline level)^15^. At the “Caution” level (21 ≤ JMA WBGT < 25), no significant differences are evident between court surfaces. At the “Warning” level (25 ≤ JMA WBGT < 28), the hard court WBGT is significantly higher than the JMA WBGT (hard WBGT, median: 27.8 °C [first quartile: 26.6 °C; third quartile: 28.7 °C] vs. JMA WBGT, 26.8 °C [first quartile: 26.3 °C; third quartile: 27.4 °C]) (*p* < 0.01). At the “Severe Warning” level (28 ≤ JMA WBGT < 31), the hard court WBGT and sand-filled artificial grass court WBGT are significantly higher than the JMA WBGT (hard WBGT, median: 30.1 °C [first quartile: 29.0 °C; third quartile: 31.2 °C]; sand-filled artificial grass WBGT, median: 30.0 °C [first quartile: 28.8 °C; third quartile: 31.3 °C]; and JMA WBGT, 29.6 °C [first quartile: 28.8 °C; third quartile: 30.3 °C]; hard WBGT, *p* < 0.01; sand-filled artificial grass WBGT, *p* < 0.01). At the “Danger” level (31 ≤ JMA WBGT), the WBGT of each surface is significantly higher than the JMA WBGT (hard WBGT, median: 32.5 °C [first quartile: 31.6 °C; third quartile: 33.5 °C]; sand-filled artificial grass WBGT, median: 32.5 °C [first quartile: 31.8 °C; third quartile: 33.5 °C]; clay WBGT, median: 32.3 °C [first quartile: 31.5 °C; third quartile: 32.9 °C]; and JMA WBGT, 31.7 °C [first quartile: 31.4 °C; third quartile: 32.1 °C]; hard WBGT, *p* < 0.01; sand-filled artificial grass WBGT, *p* < 0.01; clay WBGT, *p* < 0.01).

**Figure 3:**
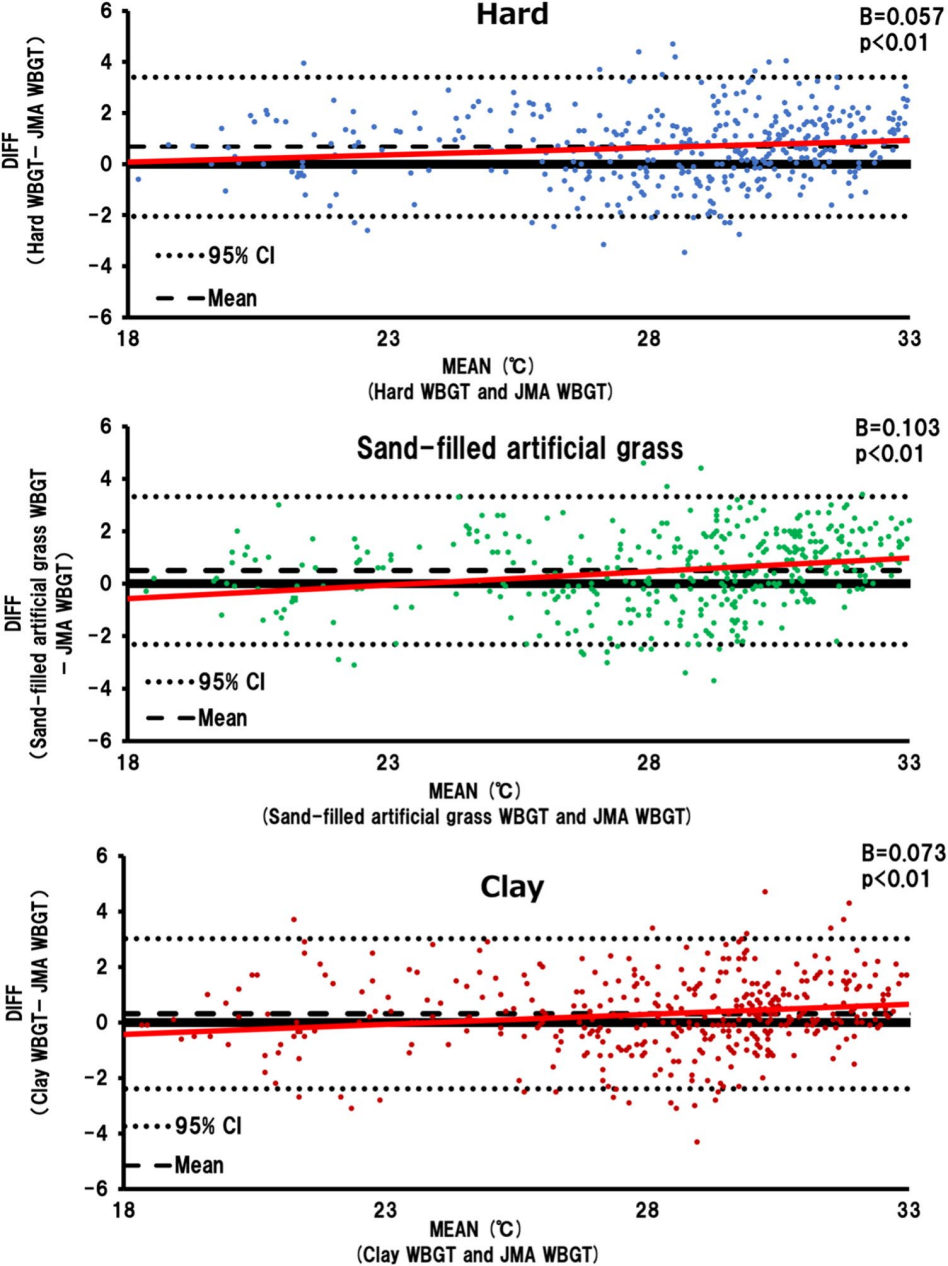
Bland–Altman plot of each court surface: Comparing the measured WBGT and JMA WBGT.

Figure 3 shows the Bland–Altman plots for the WBGT of each surface and the JMA WBGT, revealing no fixed errors (hard WBGT, 95% CI − 2.1–3.4; sand-filled artificial grass WBGT, 95% CI − 2.3–3.3; clay WBGT, 95% CI − 2.4–3.0); however, proportional errors are evident (hard WBGT, B = 0.057, *p* < 0.01; sand-filled artificial grass WBGT, B = 0.103, *p* < 0.01; clay WBGT, B = 0.073, *p* < 0.01).

### Changes in the WBGT of each surface and JMA WBGT over time

**Figure 4:**
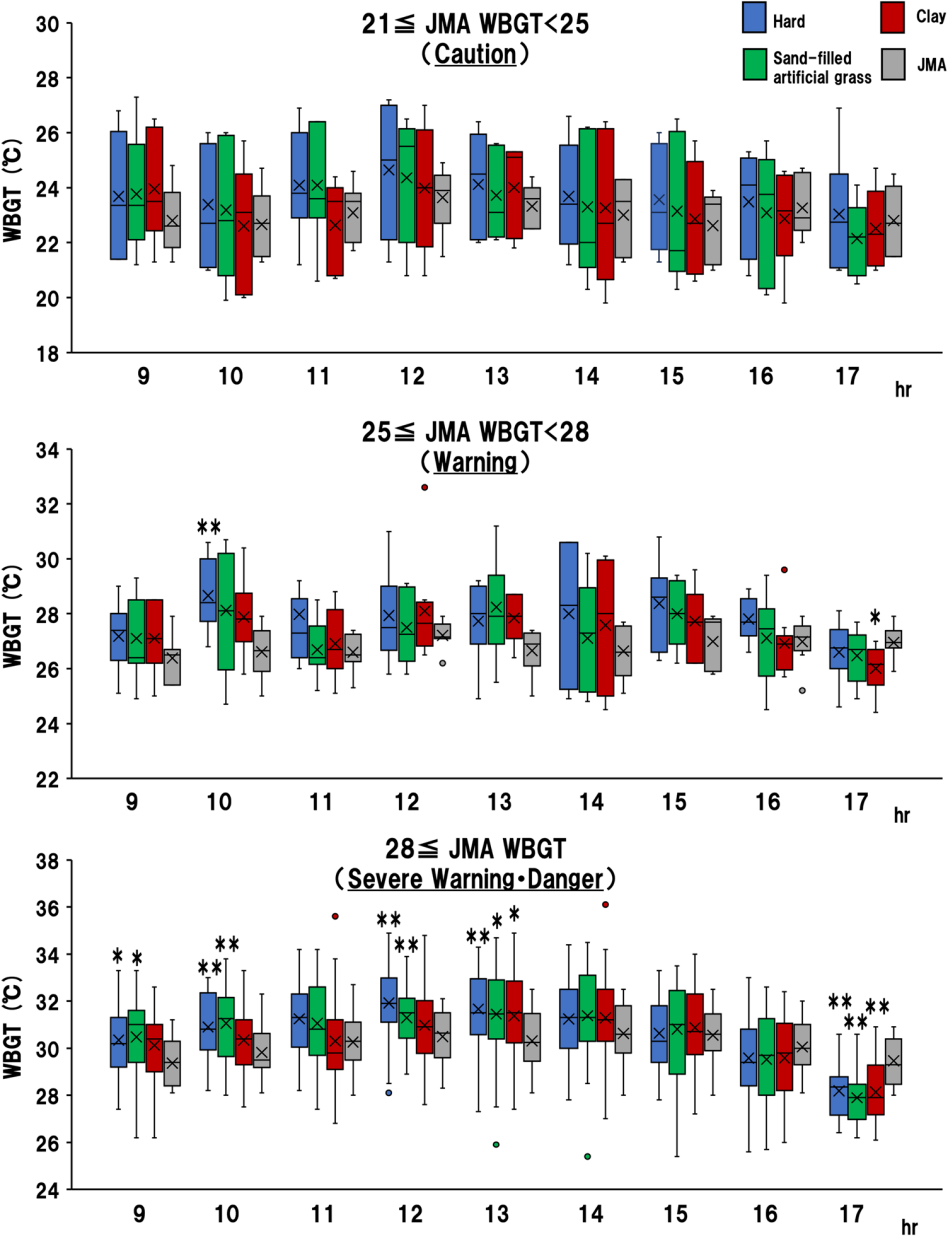
Changes over time in the JMA WBGT vs. hard WBGT by exercise guideline level. *p < 0.05 compared with JMA WBGT; **p < 0.01 compared with JMA WBGT.

Figure 4 shows the temporal variations in the WBGT of each surface and the JMA WBGT at each guideline level. At the “Warning” level (25 ≤ JMA WBGT < 28), the hard court WBGT is significantly higher than the JMA WBGT at 10:00 (median: 28.4 °C [first quartile: 27.7 °C; third quartile: 30.0 °C] vs 26.6 °C [first quartile: 25.9 °C; third quartile: 27.4 °C]) (*p* < 0.01). The JMA WBGT is significantly higher than the clay court WBGT at 17:00 (median: 27.0 °C [first quartile: 26.8 °C; third quartile: 27.4 °C] vs 26.0 °C [first quartile: 25.4 °C; third quartile: 26.7 °C]) (*p* < 0.05).

At the “Severe Warning” and “Danger” levels (28 ≤ JMA WBGT), the hard court WBGT is significantly higher than the JMA WBGT at 9:00 (median: 30.3 °C [first quartile: 29.2 °C; third quartile: 31.3 °C] vs 29.4 °C [first quartile: 28.4 °C; third quartile: 30.3 °C]), 10:00 (median: 30.9 °C [first quartile: 29.9 °C; third quartile: 32.4 °C] vs 29.8 °C [first quartile: 29.2 °C; third quartile: 30.6 °C]), 12:00 (median: 31.9 °C [first quartile: 31.1 °C; third quartile: 33.0 °C] vs 30.5 °C [first quartile: 29.6 °C; third quartile: 31.5 °C]), 13:00 (median: 31.7 °C [first quartile: 30.6 °C; third quartile: 33.0 °C] vs 30.3 °C [first quartile: 29.5 °C; third quartile: 31.5 °C]) (9:00, *p* < 0.05, 10:00, 12:00, 13:00, 17:00, *p* < 0.01).

The sand-filled artificial grass court WBGT is significantly higher than the JMA WBGT at 9:00 (median: 30.5 °C [first quartile: 29.4 °C; third quartile: 31.6 °C] vs. 29.4 °C [first quartile: 28.4 °C; third quartile: 30.3 °C]), 10:00 (median: 31.1 °C [first quartile: 29.7 °C; third quartile: 32.2 °C] vs. 29.8 °C [first quartile: 29.2 °C; third quartile: 30.6 °C]), 12:00 (median: 31.3 °C [first quartile: 30.4 °C; third quartile: 32.1 °C] vs 30.5 °C [first quartile: 29.6 °C; third quartile: 31.5 °C]), 13:00 (median: 31.4 °C [first quartile: 30.4 °C; third quartile: 32.9 °C] vs 30.3 °C [first quartile: 29.5 °C; third quartile: 31.5 °C]) (9:00, 13:00, *p* < 0.05, 10:00, 12:00, *p* < 0.01).

The clay court WBGT is significantly higher than the JMA WBGT at 13:00 (median: 31.4 °C [first quartile: 30.2 °C; third quartile: 32.9 °C] vs (median: 30.3 °C [first quartile: 29.5 °C; third quartile: 31.5 °C]) (*p* < 0.05).

The JMA WBGT is significantly higher than the WBGT of each surface at 17:00 (hard WBGT, median: 28.2 °C [first quartile: 27.2 °C; third quartile: 28.8 °C]; sand-filled artificial grass WBGT, median: 27.9 °C [first quartile: 27.0 °C; third quartile: 28.5 °C]; clay WBGT, median: 28.1 °C [first quartile: 27.2 °C; third quartile: 29.3 °C] vs 29.5 °C [first quartile: 28.5 °C; third quartile: 30.4 °C]) (*p* < 0.01).

## Discussion

In this study, more court surfaces (on-site measurements) exhibited higher values than the JMA WBGT as the WBGT increased. Hourly comparisons showed significantly higher values than the JMA WBGT from the morning to 13:00, mainly in hard courts and sand-filled artificial grass courts at the “Severe Warning” and “Danger” levels (28 ≤ JMA WBGT).

A previous study of the WBGT on different surfaces using a portable sensor-based WBGT meter reported that although there were no significant differences between surfaces, the WBGT on black-painted tennis courts (hard courts), track and field surfaces, red-painted track and field surfaces, and volleyball fields (sand) were significantly higher than those measured at the nearest station^11^. Moreover, it has been shown that the agreement of activity guidelines between on-site measurements (at each athletic field) and the values announced by the weather station decreased with increasing WBGT in the 26.6–32.0 °C range^11^.

In this study, using the natural thermometer method, more court surfaces (on-site measurements) exhibited higher values than the JMA WBGT as the WBGT increased. In particular, the hard court WBGT exhibited significantly higher values than the JMA WBGT when the WBGT was > 25 °C. The Bland–Altman plot showed a proportional error between the WBGT of each surface and the JMA WBGT—that is, as the WBGT increased, the JMA WBGT underestimated the heat environment of the WBGT of each tennis court surface, suggesting that actual on-court measurements are essential.

Furthermore, in this study, the heat environment in hard and sand-filled artificial grass courts was severe based on the low guideline levels (according to the JMA WBGT) compared to clay courts. In a previous study, the WBGT at the nearest station underestimated the heat stress on the local surface when the surface was composed of synthetic materials^11^. In this study, the hard courts and sand-filled artificial grass courts were composed of synthetic materials, and the JMA station was planted with grass to reduce heat from the ground. It could be inferred that the difference in the measurement environment (synthetic or natural materials) was a factor that caused the difference in the WBGT. This study’s long-term evaluation of the heat environment using a WBGT meter is crucial, as heat-mitigation measures from earlier guideline levels (under conditions where the JMA WBGT is expected to reach ≥ 25 °C) are required in hard courts (DecoTurf), which are widely used globally. Moreover, aggressive measures are required, particularly between 9:00 and 13:00, when the amount of solar radiation is expected to increase with an increase in the solar radiation angle.

Exertional heat stroke is the second most common cause of non-traumatic death in competitive athletes^22^. Moreover, tennis (including soft tennis) is the fourth most common sport in terms of the number of heat stroke cases in elementary, junior high, high, and vocational schools, following baseball, soccer, and endurance running^23^. Indeed, the risk of heat stroke is high in tennis tournaments that take place in hot environments, and the Australian Open has reported an increase in the number of players calling for cooling devices and requesting doctors and trainers as the WBGT increases and several matches have been abandoned because of heat-related illnesses^24^.

Several studies have reported that the number of heat stroke cases increased as the WBGT increased^25,26^. Moreover, it has been shown that the risk of heat stroke was approximately six times higher at 28–29 °C than at 25–26 °C^27^ and that deaths due to heat stroke increased rapidly at WBGTs above 28 °C^28^. Consequently, underestimation of the WBGT in the field owing to JMA observations can affect the heat protection choices and activity policies of competition organizers and athletes, leading to the risk of heat stroke and poor performance. In a survey of heat measures at regional tennis tournaments in Japan, 71.6% of tournaments “prepared ice, drinking water, parasols, stretchers or wheelchairs, and automated external defibrillators,” while only 49.3% of tournaments “measured the WBGT or temperature and humidity^29^.” Given that matches may be played under severe heat conditions (especially on hard courts compared to the WBGT values at the nearest station), coaches and tournament organizers should carefully monitor the heat environment using on-site measurements of the WBGT and prepare cooling measures in advance. It is also vital to reduce the frequency of early afternoon games and proactively explore the possibility of hosting games during the early morning, evening, or nighttime. Based on the results of this study, implementing measures (in particular, the measurement and evaluation of the heat environment in the field using WBGT meters) that consider the heat environment at each surface could prevent heat-related accidents.

This study has several limitations. Firstly, it focused solely on environmental factors contributing to heat strain without assessing the physiological load on athletes, such as water loss. Individual differences in thermoregulation, thermal comfort, and heat strain were also not considered. Previous studies have identified six factors influencing heat strain: dry-bulb temperature, black-globe temperature, wind velocity, wet-bulb temperature, metabolic rate, and clothing insulation moisture permeability^30^. A detailed analysis of environmental effects on heat stress necessitates information on four fundamental factors: air temperature, mean radiant temperature, air velocity, and absolute humidity^31^. The WBGT index provides a basic approximation of heat stress on humans. However, its limitations have been acknowledged. Budd^32^ pointed out that it does not adequately reflect the additional strain experienced when sweat evaporation is hindered by high humidity or low airflow. Additionally, Alfano et al.^33^ suggested considerations for clothing adjustments, setting limits, and adapting to changing working conditions, highlighting uncertainties in current practices. ISO7243^34^ introduced the clothing adjustment value (CAV) and effective wet bulb globe temperature (WBGT_eff_) to address these concerns by adjusting for clothing with different thermal properties than standard work clothing. When WBGT_eff_ exceeds the reference value, it recommends using the Predicted Heat Strain (PHS) index^35,36^, which integrates all six heat stress factors affecting humans^34^. The PHS model quantitatively predicts heat burden risk by incorporating air temperature, humidity, wind speed, radiant temperature, metabolic heat production, and clothing thermal resistance into a body thermal equilibrium equation^34^. Analysis using the PHS model demonstrated that mean radiant temperature significantly affects the Maximum Allowable Exposure Time (DLE), particularly noting a substantial decrease in DLE at high metabolic rates when the difference between mean radiant temperature and air temperature exceeds 10 °C^37^. Thus, utilizing the PHS model to quantitatively predict heat burden risk on each tennis court surface in the future could facilitate the development of more effective heat safety guidelines. Second, the model used to calculate the JMA WBGT is not internationally accepted. The ISO7243 standard (Ergonomics of the thermal environment assessment of heat stress using the WBGT (wet-bulb globe temperature) index) does not recommend indirect assessment of the WBGT^14^. However, in Japan, the WBGT (indirectly evaluated from meteorological data) is used to apply exercise and operational guidelines when the actual measured values at the site cannot be obtained. Consequently, we believe that it is appropriate to adopt the JMA WBGT as a comparison target for the WBGT measured at each surface, but its accuracy is not guaranteed. Third, the court used in this study was 3.8 km away from the JMA WBGT at the station, and the influence of the surrounding environment and local climate may not have been fully considered. However, setting up an observation point adjacent to the tennis court was impossible based on JMA conditions. Fourth, this study may have underestimated the infrared radiation emitted from the ground because the WBGT was measured only at 1.5 m above ground level rather than at the three heights specified in the ISO 7243 standard^14^—that is, 0.1, 1.1, and 1.7 m). However, the International Olympic Committee Consensus Statement recommends that measurements be taken at 1.2–1.5 m above the ground to consider the conditions experienced by athletes^16^. Consequently, the data obtained in this study are considered to have some utility. Fifth, the 1 °C accuracy in the WBGT assessment used in this study is only applicable to the specific climatic conditions for which the Japanese model was validated. Finally, there are various types of hard coating materials, and the results of this study are not comprehensive. Therefore, future measurements at multiple locations and heights and using different materials should be conducted to assess the impact of surfaces on the WBGT more accurately. Despite these limitations, we were able to measure the heat environment of tennis courts for approximately three months during the summer season, and our findings point out the importance of early measurements and the need for match management that considers the court surfaces and time of day (which are the strengths of this study).

## Conclusions

The WBGT was higher on many tennis court surfaces as the exercise guideline levels increased compared with the WBGT published by the JMA. Consequently, aggressive heat countermeasures should be provided for hard and sand-filled artificial grass courts at an earlier time, as more severe heat per day was evident from 9:00 to 13:00. This study also demonstrated the importance of exploring the possibility of holding games in the early morning, evening, and nighttime. Lastly, these results should highlight to tournament organizers and coaches the importance of measuring the on-site WBGT on each court surface from an early stage. On the other hand, since WBGT serves merely as an empirical indicator for assessing heat risk, future studies should aim to quantitatively predict the magnitude of deep body temperature increase and heightened sweating on each court surface. This approach should also determine allowable exposure times and identify the primary thermal factors contributing to heat risk using body heat balance models such as PHS.

## Data Availability

The data that support the findings of this study are available from the corresponding author, [I. K.], upon reasonable request.

## References

[1] Reid M, Duffield R (2014). The development of fatigue during match-play tennis. Br. J. Sports Med..

[2] Reuters. 2021. Tennis-ITF accepts players’ requests to delay start due to heat. https://www.reuters.com/lifestyle/sports/tennis-itf-accepts-players-requests-delay-start-due-heat-2021-07-28/#:~:text=TOKYO%2C%20July%2028%20(Reuters),in%20hot%20and%20humid%20conditions.

[3] Durkee, A. 2021. Tokyo Olympics: Tennis shifts later due to extreme heat after player Medvedev says he “can die” during match. *Forbes*. https://www.forbes.com/sites/alisondurkee/2021/07/28/tokyo-olympics-tennis-shifts-later-due-to-extreme-heat-after-player-medvedev-says-he-can-die-during-match/?sh=67e38ad8162b.

[4] Hernandez, J. 2021. It’s so hot in Tokyo that officials are having to reschedule the tennis matches in vol. 2021 (Ed. July, NPR.). https://www.npr.org/sections/tokyo-olympics-live-updates/2021/07/28/1021672877/its-so-hot-in-tokyo-that-officials-are-having-to-reschedule-the-tennis-matches.

[5] International Tennis Federation. 2021. Tokyo 2020: What is the extreme weather policy? https://www.itftennis.com/en/news-and-media/articles/tokyo-2020-what-is-the-extreme-weather-policy/.

[6] International Tennis Federation. n.d. ITF World Tennis Tour Juniors calendar. https://www.itftennis.com/en/tournament-calendar/world-tennis-tour-juniors-calendar/?categories=All&startdate=2022.

[7] Japan Tennis Association. n.d. JTA WEB tennis calendar latest calendar for 2022 junior tournaments. https://www.jta-tennis.or.jp/Portals/0/resources/tournaments/pdf/calendar/2022/cal2022jr.pdf.

[8] Kovacs MS (2006). Hydration and temperature in tennis–A practical review. J. Sports Sci. Med..

[9] Kosaka E (2018). Microclimate variation and estimated heat stress of runners in the 2020 Tokyo Olympic marathon. Atmosphere.

[10] Grundstein A, Cooper E (2020). Comparison of WBGTs over different surfaces within an athletic complex. Medicina.

[11] Pryor JL, Pryor RR, Grundstein A, Casa DJ (2017). The heat strain of various athletic surfaces: A comparison between observed and modeled wet-bulb globe temperatures. J. Athl. Train..

[12] Yaglou CP, Minard CD (1957). Control of heat casualties at military training. Am. Med. Ass. Arch. Indust. Health.

[13] Ministry of the Environment. 2020. Heat illness prevention guidelines for summer events. https://www.wbgt.env.go.jp/pdf/gline/heatillness_guideline_full.pdf.

[14] ISO 7243. 1989. Hot environments—Estimation of the heat stress on working man, based on the WBGT-index (wet bulb globe temperature).

[15] Kawahara, T. *et al. Guidebook for prevention of heat disorders in sports activities*, 5th edn. https://www.japan-sports.or.jp/Portals/0/data/supoken/doc/heatstroke/heatstroke_0531.pdf. (in Japanese) (Japan Sports Assoc., 1994).

[16] Racinais (2023). IOC consensus statement on recommendations and regulations for sport events in the heat. Br. J. Sports Med..

[17] Japan Tennis Association. *Application of heat rules and medical rules in tennis competitions in heat*, Rev. Edn. http://medical.jta-tennis.or.jp/tennismedical/jigyou/pdf/jta-010-j.pdf. (in Japanese) (2020).

[18] Ministry of the Environment. 2022. Heat illness prevention information. https://www.wbgt.env.go.jp/data_service.php.

[19] Ono M, Tonouchi M (2014). Estimation of wet-bulb globe temperature using generally measured meteorological indices. Jpn. J. Biometeorol..

[20] Iribarne Godson. Atmospheric Thermodynamics. 3rd Edn. *D. Reidel*, 259 (1981).

[21] Bland JM, Altman DG (1986). Statistical methods for assessing agreement between two methods of clinical measurement. Lancet.

[22] Maron BJ, Doerer JJ, Haas TS, Tierney DM, Mueller FO (2009). Sudden deaths in young competitive athletes: Analysis of 1866 deaths in the United States, 1980–2006. Circulation.

[23] Japan Sports Council. 2022. Disasters under school administration. https://www.jpnsport.go.jp/anzen/Portals/0/anzen/anzen_school/R4_gakko_kanrika_saigai/R4-08.pdf (in Japanese).

[24] Smith MT, Reid M, Kovalchik S, Woods TO, Duffield R (2018). Heat stress incident prevalence and tennis matchplay performance at the Australian Open. J. Sci. Med. Sport.

[25] Iwashita G (2018). Risk of heatstroke determined using data on accidents that occurred during club activities at secondary/high schools: relationship between outdoor climate and accidents at schools (Part 4). Jpn. Archit. Rev..

[26] DeMartini JK (2014). Environmental conditions and the occurrence of exertional heat illnesses and exertional heat stroke at the Falmouth Road Race. J. Athl. Train..

[27] Cooper ER (2016). Exertional heat illness in American football players: When is the risk greatest?. J. Athl. Train..

[28] Nakai S (2007). Proposal of new guidelines for prevention of heat disorders during sports and daily activities based on age, clothing and heat acclimatization. Jpn. J. Phys. Fit. Sports Med..

[29] Japan Tennis Association. Report on the Tennis Environment in Japan. https://www.jta-tennis.or.jp/Portals/0/resources/JTA/pdf/information/population/population_h30_jpn.pdf. (in Japanese).

[30] Fanger PO (1970). Thermal comfort.

[31] ISO 7726. 2002. Ergonomics of the thermal environment—instruments for measuring physical quantities.

[32] Budd GM (2008). Wet-bulb globe temperature (WBGT)–itshistory and its limitations. J. Sci. Med. Sport.

[33] d’Ambrosio Alfano FR, Malchaire J, Palella BI, Riccio G (2014). The WBGT index revisited after 60 years of use. Ann. Occup. Hyg..

[34] ISO 7243. 2017. Ergonomics of the thermal environment—Assessment of heat stress using the WBGT (wet bulb globe temperature) index.

[35] Malchaire JB (2001). Development and validation of the predicted heat strain model. Ann. Occup. Hyg..

[36] ISO 7933. 2023. Ergonomics of the thermal environment—Analytical determination and interpretation of heat stress using calculation of the predicted heat strain.

[37] Palella BI, Quaranta F, Riccio G (2016). On the management and prevention of heat stress for crews onboard ships. Ocean Eng..

